# Structural characterization of the human organic anion transporter OATP2B1

**DOI:** 10.1038/s41421-026-00938-w

**Published:** 2026-10-31

**Authors:** Jiahui Chen, Lanmin Wang, Min Zhao, Shijian Feng, Wencheng Liu, Lei Xiang, Liang Zhou, Kunjie Wang, Bowen Ke, Jie Yu, Dongxue Yang

**Affiliations:** 1 Department of Urology, Institute of Urology (Laboratory of Reconstructive Urology), West China Hospital, Sichuan University, Chengdu, Sichuan China; 2 Interdisciplinary Research Center on Biology and Chemistry, Shanghai Institute of Organic Chemistry, Chinese Academy of Sciences, Shanghai, China; 3 University of Chinese Academy of Sciences, Beijing, China; 4 Tianfu Jincheng Laboratory, Chengdu, Sichuan China; 5 Department of Anesthesiology, Laboratory of Anesthesia and Critical Care Medicine, West China Hospital, Sichuan University, Chengdu, Sichuan China; 6 Frontiers Science Center for Disease-Related Molecular Network, West China Hospital, Sichuan University, Chengdu, Sichuan China; 7 Shanghai Key Laboratory of Aging Studies, Shanghai, China

**Keywords:** Electron microscopy, Cell signalling

Dear Editor,

Organic anion transporting polypeptides (OATPs), encoded by *SLC21*/*SLCO* genes, are essential influx transporters responsible for the cellular uptake of diverse endogenous molecules and clinical drugs^1–3^. The OATP family comprises six subfamilies with distinct tissue distribution and substrate specificity, among which liver-enriched OATP1B1/1B3 and widely distributed OATP2B1 are the most pharmacologically critical isoforms^4^. Unlike OATP1B1/1B3 that primarily mediate hepatic drug clearance, OATP2B1 regulates both intestinal drug absorption and hepatic extraction, serving as a key mediator of food–drug and drug–drug interactions (DDIs)^5,6^. Accumulating evidence indicates that OATP2B1 inhibition contributes to unexplained alterations in systemic drug exposure beyond OATP1B1/1B3 modulation, highlighting its unique pharmacological importance^7^. Moreover, genetic variations in *SLCO2B1* alter drug transport efficiency, leading to interindividual differences in drug response and adverse drug reactions^8,9^. Despite its functional significance, the lack of high-resolution structural information of OATP2B1 has hindered the mechanistic understanding of its substrate recognition, specificity, and conformational transport dynamics.

OATP2B1 exhibits a broader tissue distribution than other OATP members, including in the small intestine, liver, blood–brain barrier, heart, and placenta^3^, and transports a broad spectrum of substrates ranging from endogenous steroid conjugates to clinical drugs such as atorvastatin, rosuvastatin, and erlotinib^10^. As the predominant intestinal OATP isoform, OATP2B1 dominates the intestinal absorption of substrate drugs and is readily inhibited by fruit juices and small-molecule therapeutics, causing clinically significant DDIs^7,10,11^. Previous structural studies of OATPs have focused on OATP1B1 and OATP1B3, which possess conserved aromatic residue-enriched binding pockets for substrate recognition^12,13^. In contrast, the structural basis underlying OATP2B1’s distinct substrate preference, broad ligand spectrum, and unique pharmacological properties remains elusive. Here, we report four cryo-electron microscopy (cryo-EM) structures of human OATP2B1 in the apo state and in complex with a fluorescent probe (4′,5′-dibromofluorescein, DBF), an endogenous substrate (pregnenolone sulfate, PregS), and a clinical drug (erlotinib), with resolutions ranging from 3.6 Å to 3.9 Å (Supplementary Figs. S1–S6 and Table S1). Combined with cell-based functional assays and mutagenesis validation, our structures uncover the precise ligand binding modes, key functional residues, and conformational switching mechanism of OATP2B1, providing a mechanistic framework for understanding OATP2B1-mediated drug transport and DDI risks.

To elucidate the impact of diverse ligands on the function of human OATP2B1, we first characterized the transport and inhibitory properties of OATP2B1 ligands using HEK293/OATP2B1 cell-based assays. The fluorescent substrate DBF exhibited specific OATP2B1-mediated uptake with a *K*_m_ value of 2.66 µM (Supplementary Fig. S7a–d), confirming its high-affinity binding to the transporter. To further investigate how various ligands modulate the activity of OATP2B1, we examined the inhibitory effects of PregS (a sulfated steroid essential for neuronal development and cognitive functions, and a potential endogenous substrate of OATP2B1) and erlotinib (a U.S. FDA-approved anticancer drug for non-small cell lung cancer and pancreatic cancer, and a substrate for OATP2B1) on DBF uptake. Functional inhibition assays showed that PregS and erlotinib inhibited DBF uptake with IC_50_ values of 2.71 µM and 0.16 µM (Supplementary Fig. S7e, f), respectively, consistent with previous pharmacological records. We then solved the cryo-EM structures of OATP2B1 in apo, DBF-bound, PregS-bound, and erlotinib-bound states at resolutions of 3.6–3.9 Å. All resolved structures adopt a canonical inward-open conformation, consistent with the conserved structural feature of OATP family transporters (Fig. 1a; Supplementary Figs. S6, S8a).

**Fig. 1:**
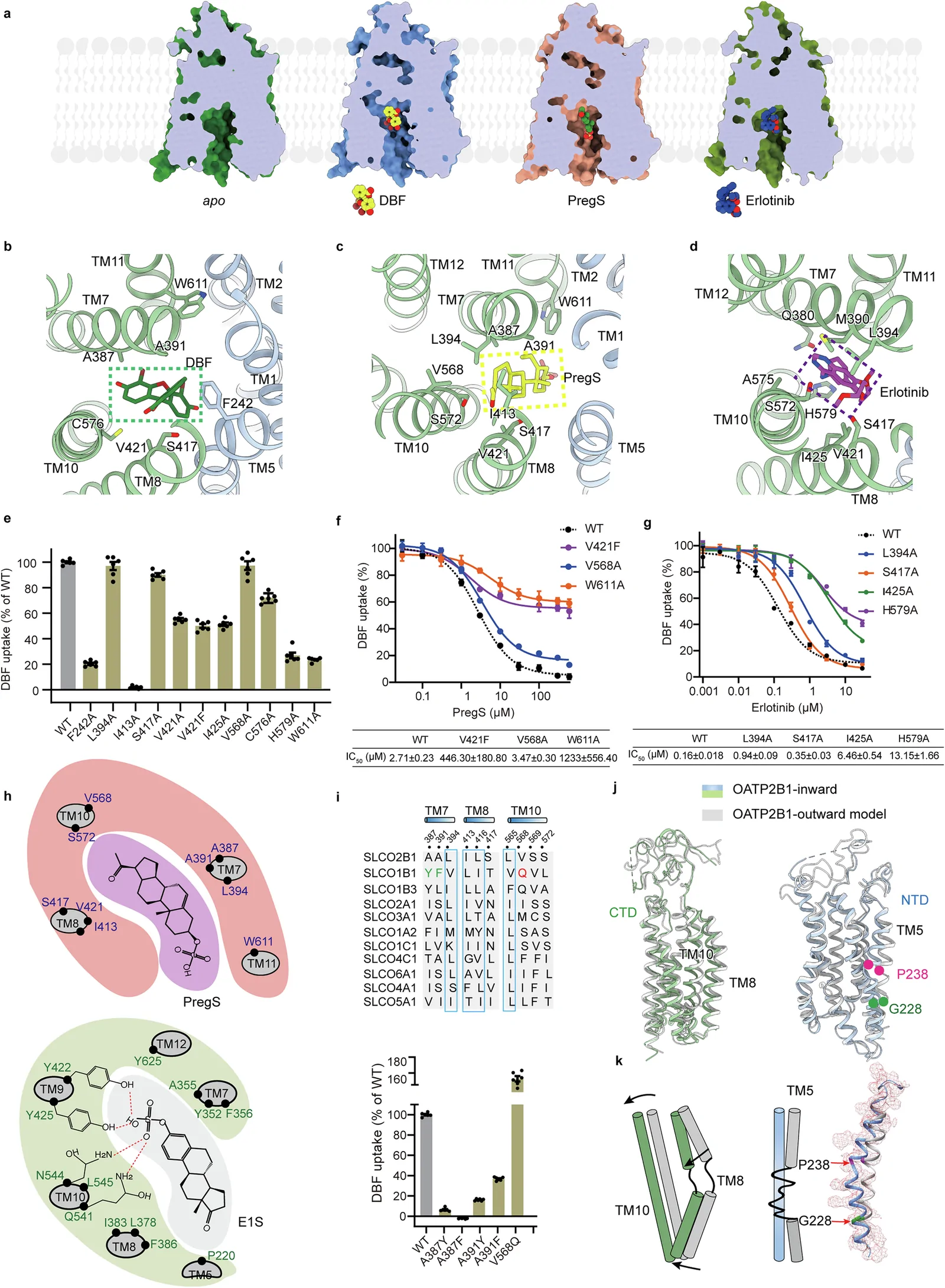
Cryo-EM structures and substrate binding mechanism of human OATP2B1. **a** Slab view of the intracellular cavities of OATP2B1. **b**–**d** Extracellular views of the substrate-binding sites in the OATP2B1–DBF (**b**), OATP2B1–PregS (**c**), and OATP2B1–erlotinib (**d**) structures. **e** DBF uptake activity assay showing reduced activity of OATP2B1 mutants in residues within major and minor pockets. The activity was normalized to that of the wild-type (WT) transporter (means ± SEM, *n*  =  6). **f** Concentration–inhibition curve of DBF uptake mediated by PregS in WT OATP2B1 and its mutants (means ± SEM, *n*  =  3). **g** Concentration–inhibition curve of DBF uptake mediated by erlotinib in WT OATP2B1 and its mutants (means ± SEM, *n*  =  3). **h** Interactions between PregS and OATP2B1, and between E1S and OATP1B1. **i** Upper, sequence alignment of residues in the binding sites across OATP family members; lower, cell-based transport assays for WT OATP2B1 and its mutants (means ± SEM, *n*  =  6). **j** Homology model of the outward-facing OATP2B1 superimposed on the inward-facing conformation. **k** The TM conformational changes in **j**.

Overall structural analysis revealed that OATP2B1 shares low sequence identity (30%) with OATP1B1^13^ (PDB: 8HND, root mean square deviation (RMSD) = 1.654) and OATP1B3^12^ (PDB: 8PG0, RMSD = 1.652) (Supplementary Figs. S8a, S9), but exhibits a conserved major facilitator superfamily (MFS) architecture, consisting of 12 transmembrane helices (TMs) divided into N-terminal (TM1–TM6) and C-terminal (TM7–TM12) pseudo-symmetric bundles (Supplementary Fig. S6c). The intracellular helix h1 connects the two domains, while the extracellular region contains multiple disulfide-stabilized loops, including a conserved Kazal domain in ECL5, whose functional role in the OATP family remains uncharacterized. In each ligand-bound cryo-EM map, a prominent density corresponding to a single ligand molecule was observed within a funnel-shaped pocket. The ligand models were docked into the EM densities and validated by molecular dynamics (MD) simulations (Supplementary Fig. S5). All ligands occupy the funnel-shaped central pocket located at the interface between the N-terminal domain (NTD) and the C-terminal domain (CTD), formed by residues from TM1, TM5, TM7, TM8, TM10, and TM11 (Supplementary Fig. S6b, c). Distinct from OATP1B1/1B3, the OATP2B1 binding pocket lacks the conserved aromatic core residues, with the corresponding positions occupied by aliphatic residues A387, A391, and V421, which form extensive interactions with the ligands (Supplementary Fig. S9).

Ligand-specific binding modes were clearly defined in our complex structures. In the DBF-bound structure, the rigid planar aromatic scaffold of DBF is stabilized by hydrophobic packing and van der Waals interactions with A391, V421, F242, and W611 (Fig. 1b; Supplementary Fig. S8b, c). Mutagenesis assays confirmed that F242A, V421A, V421F, and W611A mutations significantly impaired DBF uptake, verifying the critical roles of these residues (Fig. 1e; Supplementary Fig. S8f). For endogenous PregS, the negatively charged sulfate group forms stable salt bridges with H579 (TM10) and R607 (TM11), while its rigid steroid backbone engages in extensive hydrophobic interactions with aliphatic residues including A387, A391, L394 (TM7), V421 (TM8), V568, S572 (TM10), and W611 (TM11) (Fig. 1c; Supplementary Fig. S8d). PregS binding was markedly disrupted by V421F and W611A substitutions, validating their functional importance (Fig. 1f; Supplementary Fig. S8f). The clinical drug erlotinib displays high conformational flexibility, with its quinazoline core anchored between TM7 and TM10 and its flexible side chains extending to interact with M390 and L394 (TM7), as well as A575 (TM10). The two 2-methoxyethoxy side chains of erlotinib can independently bend and extend along different clefts: one inserts between TM7 and TM8 to interact with S417 and S572, while the other projects outward to contact V421 and Q380. The terminal phenyl ring also undergoes rotational adjustment to form polar contacts with Q380 (TM7) and H579 (TM10). This high conformational flexibility enables erlotinib to adapt to the irregular, extended binding pocket of OATP2B1 and establish multi-point interactions across several transmembrane helices (Fig. 1d; Supplementary Fig. S8e). Alanine mutagenesis of L394, S417, I425, and H579 significantly increased erlotinib IC_50_ values, confirming these residues as key determinants of drug recognition (Fig. 1g; Supplementary Fig. S8f).

Structural comparison between OATP2B1 and OATP1B1 uncovered the molecular basis for their divergent substrate specificities despite overlapping steroid conjugate recognition (Supplementary Fig. S10a). OATP1B1 relies on conserved aromatic residues Y352, F356, F386 and polar residues N544, Q541 to form aromatic stacking and hydrogen bonds with steroid substrates such as estrone 3-sulfate (E1S). In contrast, PregS binding in OATP2B1 is predominantly driven by hydrophobic interactions via aliphatic residues, with no conserved polar sulfate-binding microenvironment (Fig. 1h). To assess the functional significance of these differences, mutagenesis studies were performed and further validated this mechanism of specificity: substitution of OATP2B1 aliphatic residues A387 and A391 with aromatic residues (tyrosine or phenylalanine) reduced transport activity, whereas V568Q polar substitution enhanced substrate uptake, suggesting that the native aliphatic environment is optimized for hydrophobic substrate interaction. Conversely, mutating V568 to glutamine (a charged residue) enhanced DBF uptake by OATP2B1, indicating that introducing a polar interaction at position V568 can modulate substrate specificity, potentially mimicking the sulfate-binding environment of OATP1B1 (Fig. 1i).

To further investigate the conformational transitions of OATP2B1, we generated an outward-open structural model using the outward-open structure of OATP1B1 as a template. Consistent with the canonical MFS rocker-switch model, the NTD and CTD undergo rigid-body rotation to alternately expose the central pocket to extracellular and intracellular environments (Fig. 1j). A prominent conformational hallmark is the partial unwinding of TM5 at the conserved GlyX9Pro antiporter motif. This structural motif functions as a molecular hinge that enables large-scale domain rearrangements upon substrate translocation, representing a mechanistic feature conserved among the OATP^14^ and NSS families^15^ (Fig. 1j, k; Supplementary Fig. S10b). Mutagenesis targeting the conserved glycine and proline residues within this region — including variants G228A, G228C, G228S, P238A and P238C — uniformly diminished substrate transport capacity of the transporter (Supplementary Fig. S10c), confirming that this conserved motif is indispensable for proper conformational switching. Localized shifts of TM8 and TM10 further fine-tune pocket conformation to accommodate ligand binding and release (Supplementary Fig. S10b–d).

In summary, our study presents the first high-resolution structural atlas of human OATP2B1 in multiple functional states, revealing distinct ligand binding modes for endogenous substrates and clinical drugs. The unique aliphatic microenvironment of the OATP2B1 binding pocket defines its broad substrate spectrum and differentiates its functional properties from liver-specific OATP1B1/1B3. The identified key functional residues and conformational switching mechanism provide definitive mechanistic insights into OATP2B1-mediated intestinal and hepatic drug transport, clinically relevant DDIs, and interindividual drug response variability. These findings establish a critical structural foundation for precise DDI prediction, personalized drug administration, and rational drug design targeting OATP2B1.

## Supplementary information

Supplementary Information

## Data Availability

All data needed to evaluate the conclusions of this paper are provided in the paper and/or the Supplementary Information. The cryo-EM maps and associated atomic coordinate models have been deposited in the wwPDB OneDep System under PDB IDs and EMDB accession codes of 9VER and EMD-65015 for apo-OATP2B1, 9VES and EMD-65016 for DBF-bound OATP2B1, 9VET and EMD-65017 for PregS-bound OATP2B1, 9VEV and EMD-65019 for erlotinib-bound OATP2B1.
