# Supplementary material for: Structural characterization of the human organic anion transporter OATP2B1
Source: Cell Discov. 2026 Oct 31;12:77. doi: 10.1038/s41421-026-00938-w (PMC13723507; doi:10.1038/s41421-026-00938-w)
Supplement: Supplementary file 1 [file 41421_2026_938_MOESM1_ESM.pdf]

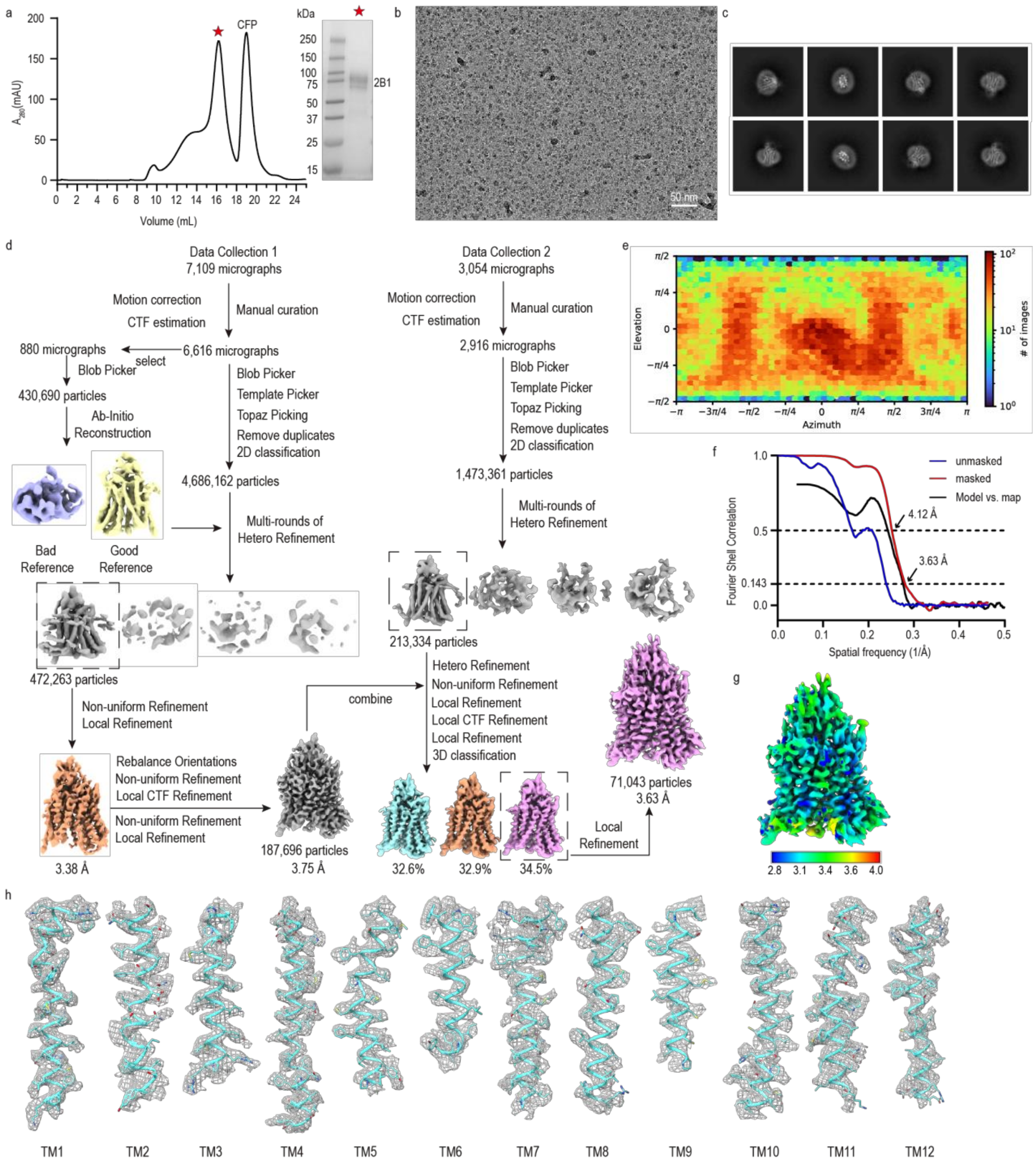

Supplementary Fig. S1 Protein purification and Cryo-EM reconstruction of OATP2B1-apo. **a.** Size-exclusion chromatography of purified human OATP2B1 protein. The fractions were pooled for cryo-EM sample preparation and SDS-PAGE gel analysis to assess purity and molecular weight. **b.** A representative cryo-EM micrograph. Scale bar: 50 nm. **c.** Representative 2D class averages generated from particles for OATP2B1-apo map. **d.** Data processing flowchart of OATP2B1-apo. Further details can be found in Methods. The contour level of final map is 0.3. **e.** Angular sampling of the final reconstruction, generated from cryoSPARC. **f.** Gold standard Fourier Shell Correlations (FSC) curves are shown for the unmasked map (blue), masked map

(red), and model-to-map correlation (black). Resolutions corresponding to FSC thresholds of 0.5 and 0.143 are indicated on the curves, providing quantitative measures of map quality and model-to-map agreement. **g.** Local resolution map of OATP2B1-apo, colored according to the estimated value ranging from 2.8 to 4.0 Å. **h.** The cryo-EM densities of transmembrane helices (TM) 1-12 in OATP2B1-apo, contoured at 0.2.

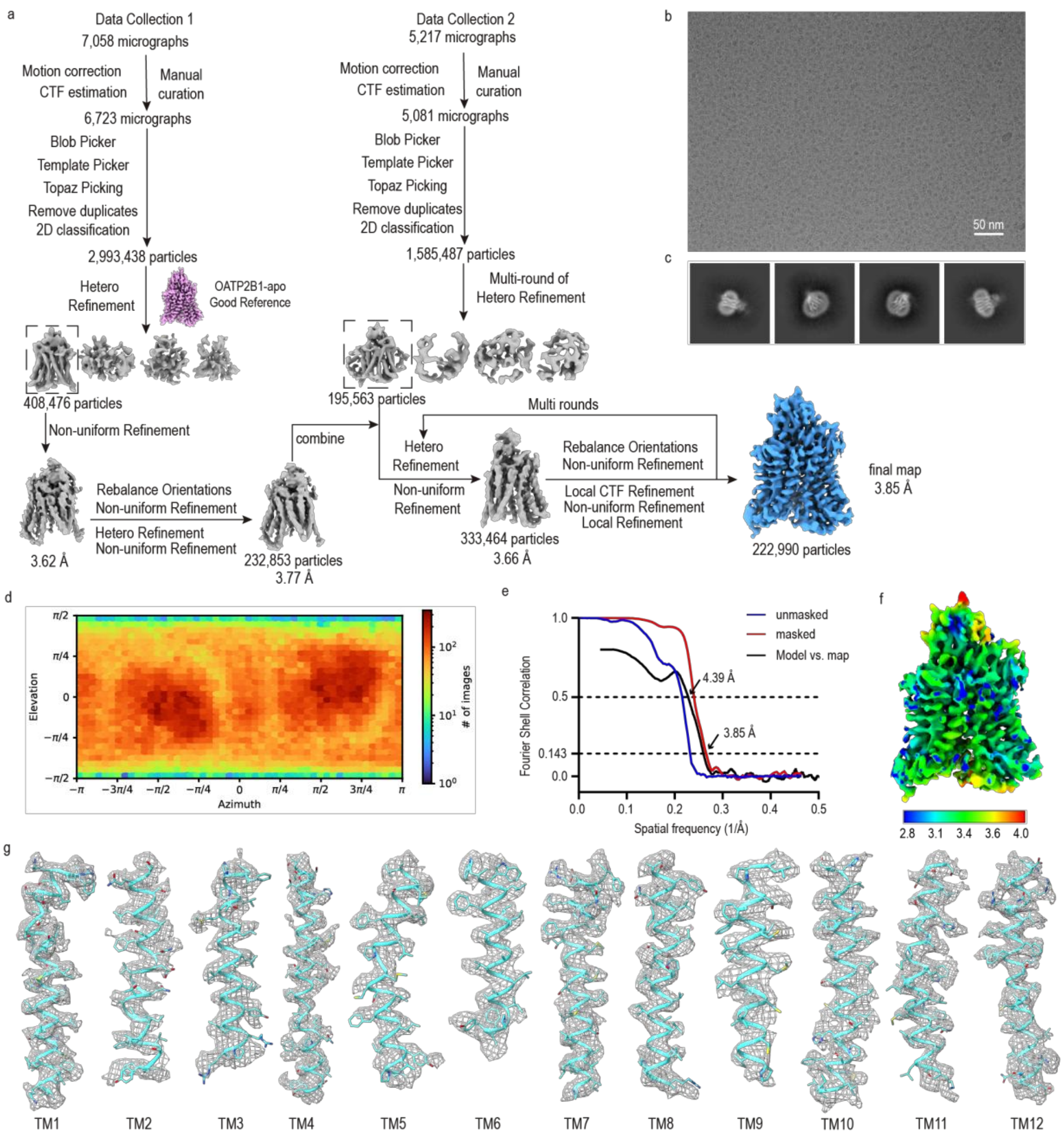

Supplementary Fig. S2 Cryo-EM reconstruction of OATP2B1-DBF. a. A schematic flowchart of OATP2B1-DBF. Detailed methodological procedures are described in the Methods section. The contour level of final map is 0.2. **b.** A representative cryo-EM micrograph. Scale bar: 50 nm. **c.** Representative 2D class averages generated from particles for OATP2B1-DBF map. **d.** Angular sampling of the final reconstruction, generated from cryoSPARC. **e.** Gold standard Fourier Shell Correlations (FSC) curves of unmasked map (blue), masked map (red) and model versus map (black), marked with resolutions corresponding to FSC = 0.5 and 0.143. **f.** Cryo-EM density map colored by local resolution estimation for OATP2B1-DBF complex, ranging from 2.8 to 4.0 Å. **g.** The cryo-EM densities of transmembrane helices in OATP2B1-DBF complex, contoured at 0.15.

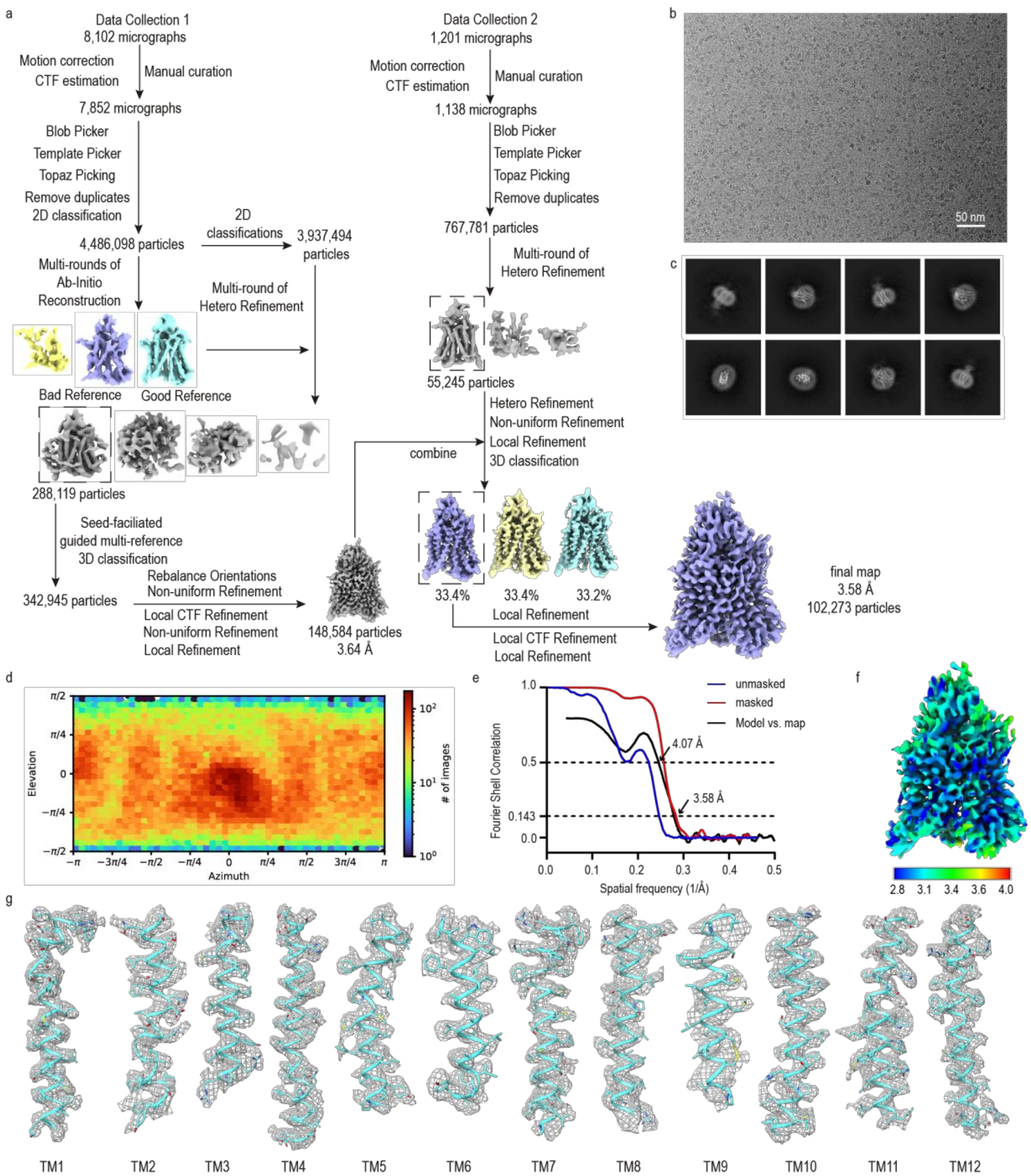

Supplementary Fig. S3 Cryo-EM reconstruction of OATP2B1-PregS. a. Data processing flowchart of OATP2B1-PregS. Further details can be found in Methods. The contour level of final map is 0.3. **b.** A representative cryo-EM micrograph. Scale bar: 50 nm. **c.** Representative 2D class averages generated from particles for OATP2B1-PregS map. **d.** Angular sampling of the final reconstruction, generated from cryoSPARC. **e.** Gold standard Fourier Shell Correlations (FSC) curves of unmasked map (blue), masked map (red) and model versus map (black), marked with resolutions corresponding to FSC = 0.5 and 0.143. **f.** Local resolution map of OATP2B1-PregS, colored according to the estimated value ranging from 2.8 to 4.0 Å. **g.** The cryo-EM densities segments for TM1-TM12 of OATP2B1-PregS complex, contoured at 0.25.

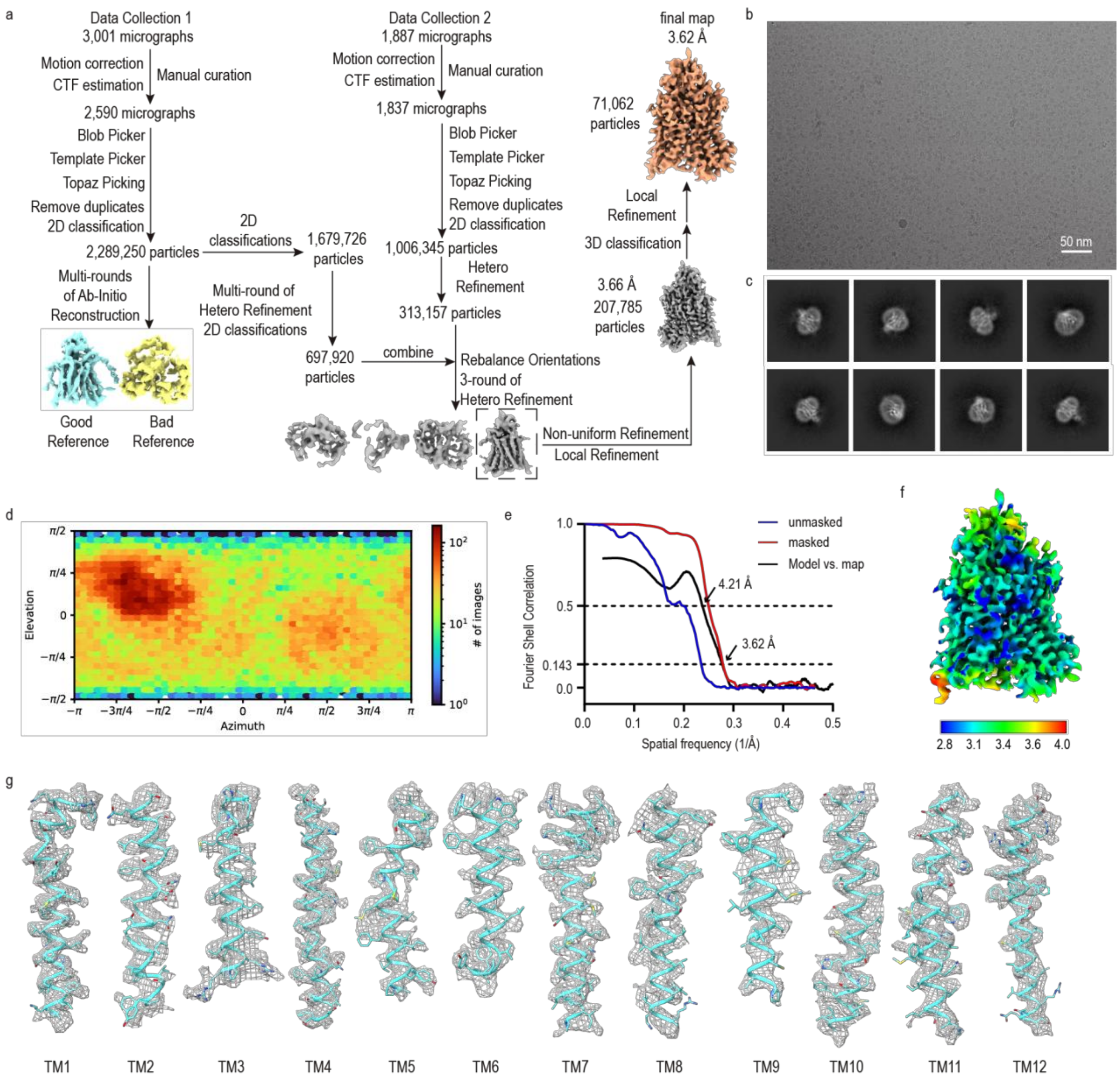

Supplementary Fig. S4 Cryo-EM reconstruction of OATP2B1-Erlotinib. a. Data processing flowchart of OATP2B1-Erlotinib. Further details can be found in Methods. The contour level of final map is 0.3. **b.** A representative cryo-EM micrograph. Scale bar: 50 nm. **c.** Representative 2D class averages generated from particles for OATP2B1-Erlotinib map. **d.** Angular sampling of the final reconstruction, generated from cryoSPARC. **e.** Gold standard Fourier Shell Correlations (FSC) curves of unmasked map (blue), masked map (red) and model versus map (black), marked with resolutions corresponding to FSC = 0.5 and 0.143. **f.** Local resolution distribution of the final map of OATP2B1-Erlotinib, colored according to the estimated value ranging from 2.8 to 4.0 Å. **g.** The cryo-EM densities of TM1-12 in OATP2B1-Erlotinib, contoured at 0.3.

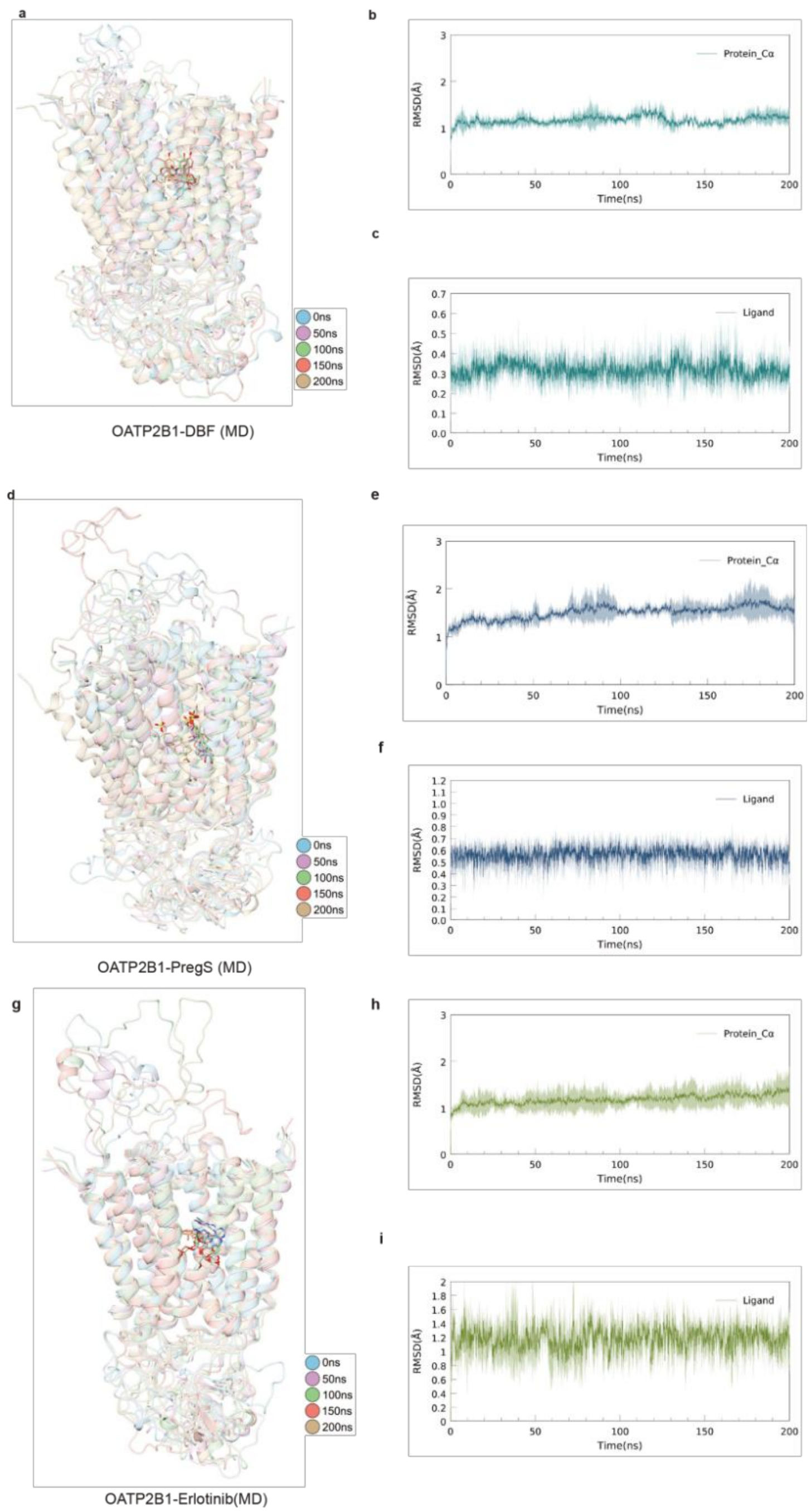

Supplementary Fig. S5 Molecular dynamics simulations of OATP2B1 with DBF, Erlotinib, and PregS over 200 ns. Snapshots extracted at regular time intervals during the simulations were superimposed to depict the conformational stability of the complexes: OATP2B1–DBF (a), OATP2B1–PregS (d), and OATP2B1–Erlotinib (g). Corresponding RMSD profiles of the protein and ligand are presented for each system: OATP2B1–DBF (b, c), OATP2B1–PregS (e, f), and OATP2B1–Erlotinib (h, i).

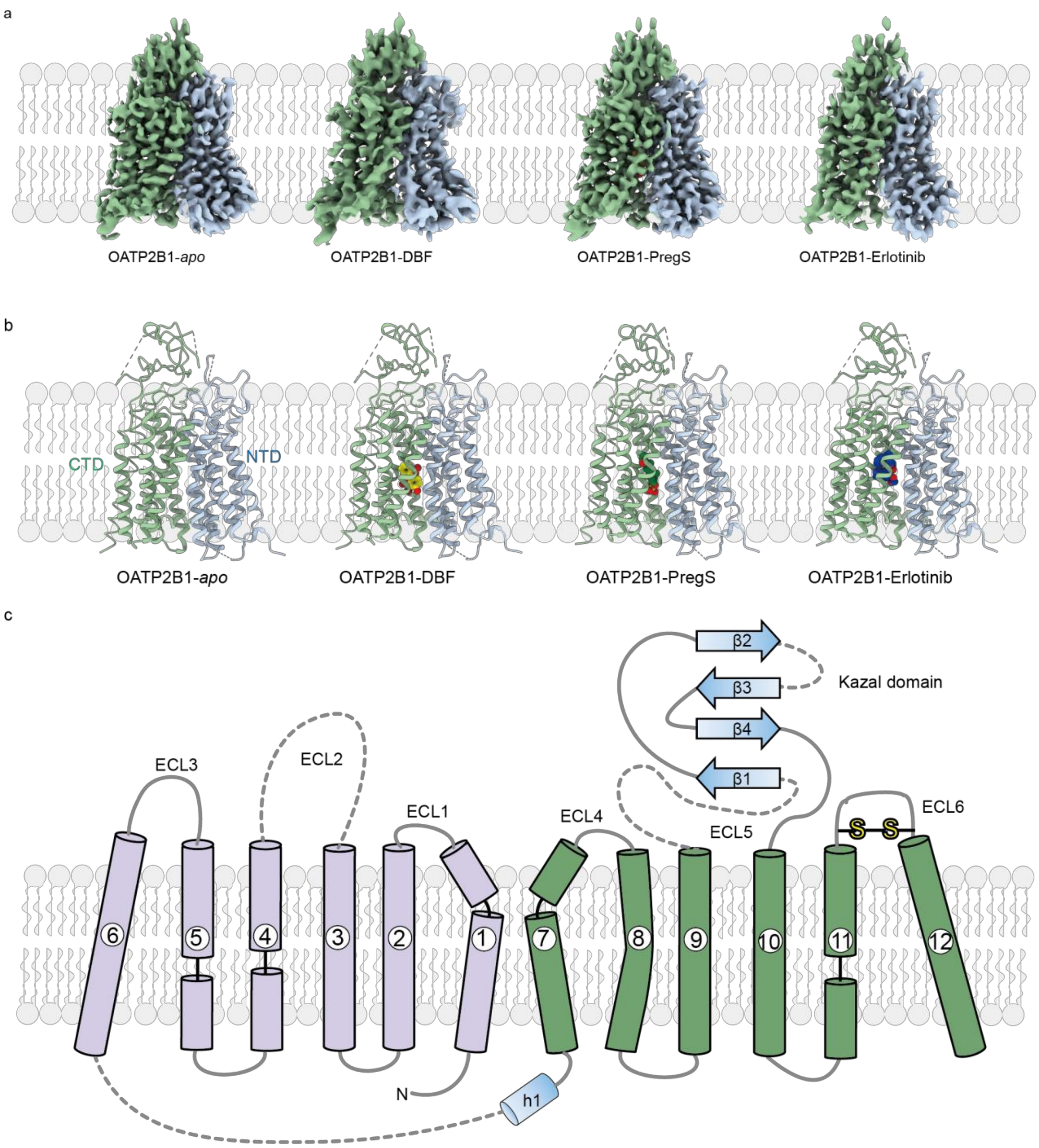

Supplementary Fig. S6 Architecture of OATP2B1 in its inward-open conformation. a. Cryo-EM density maps of OATP2B1 shown in its apo and ligand-bound forms. The N-terminal domain (NTD) and C-terminal domain (CTD) are colored light blue and green, respectively. **b.** Atomic models of OATP2B1 in its apo and ligand-bound forms, with the NTD and CTD colored light blue and green, respectively. **c.** Schematic diagram of the secondary structures of OATP2B1.

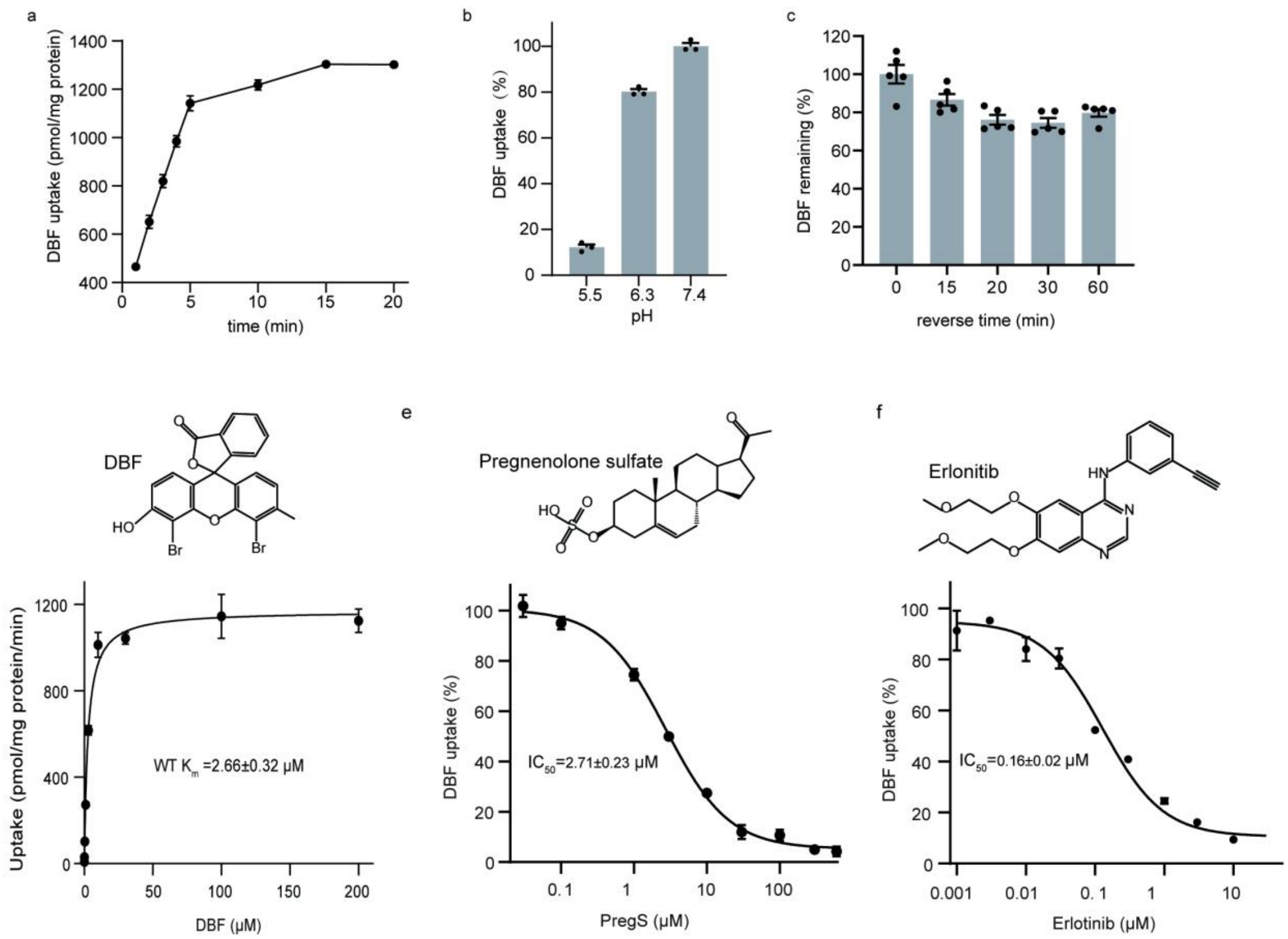

Supplementary Fig. S7 Functional characterization of OATP2B1. a. Time-dependent uptake of DBF by OATP2B1. **b.** pH dependence of DBF uptake. **c.** Measurement of DBF reverse transport by OATP2B1. **d.** Cell-based transport assay for wild-type (WT) human OATP2B1 with DBF, Km for OATP2B1 is 2.6 μM. **e.** Plots of competition binding of PregS against DBF. **f.** Competition binding of erlotinib against DBF. Data are means with SEM of three independent experiments

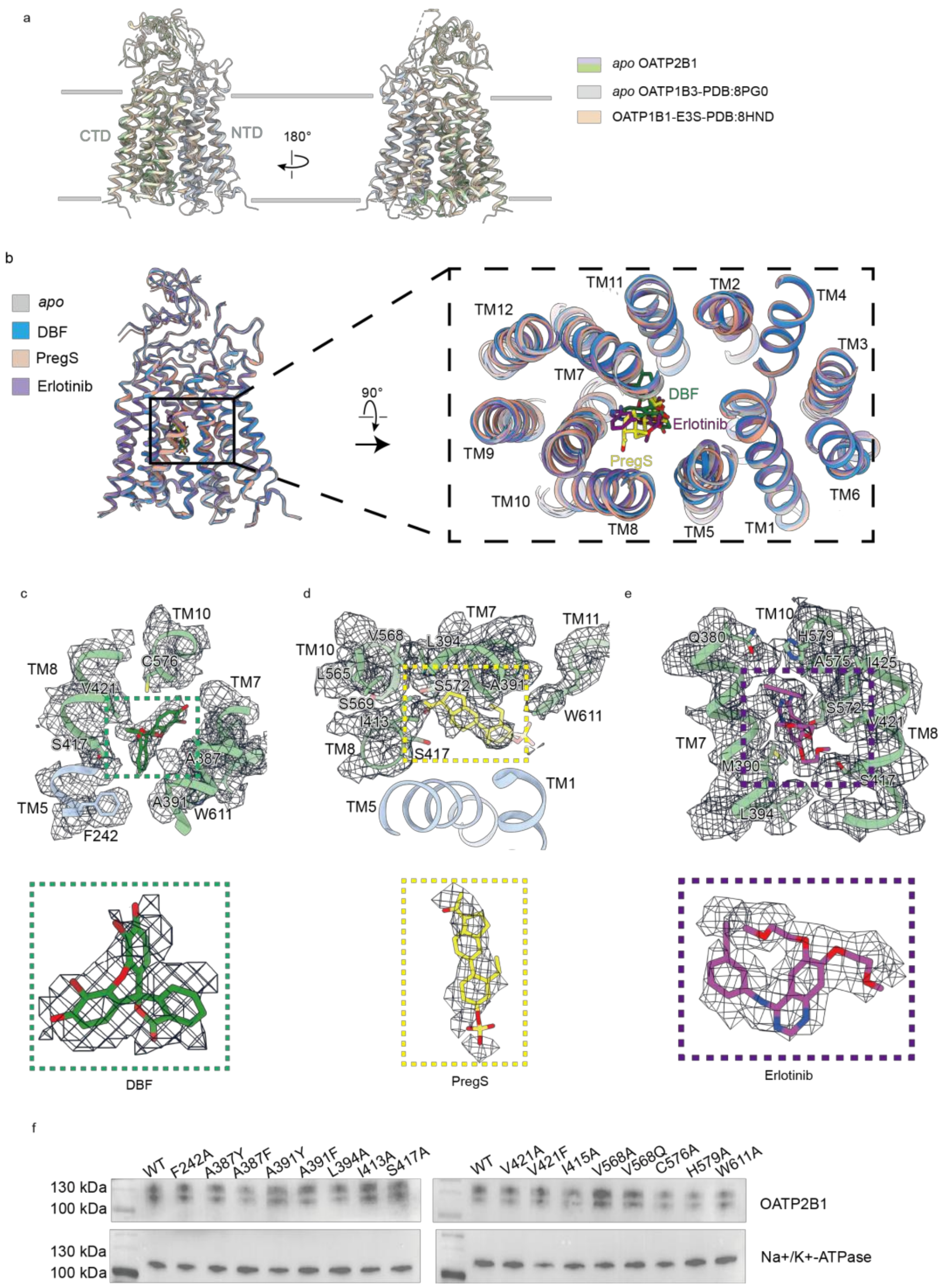

Supplementary Fig. S8 Comparison of OATP2B1 with OATP1B1 and OATP1B3, and ligand binding pockets. **a.** Superposition of apo OATP2B1 (purple and green) with OATP1B1 (PDB code: 8HND, orange) and OATP1B3 (PDB code: 8PG0, grey). **b.** Superimposition of inward-open structures of OATP2B1-apo/DBF/Pregs/erlotinib. **c-e.** Local ligand density representations for substrate binding sites of OATP2B1–DBF (c), OATP2B1–Pregs (d) and OATP2B1–erlotinib (e). **f.** Western blot analysis of cell-surface expression levels of wild-type (WT) and mutant OATP2B1, used for normalization of DBF uptake activity.

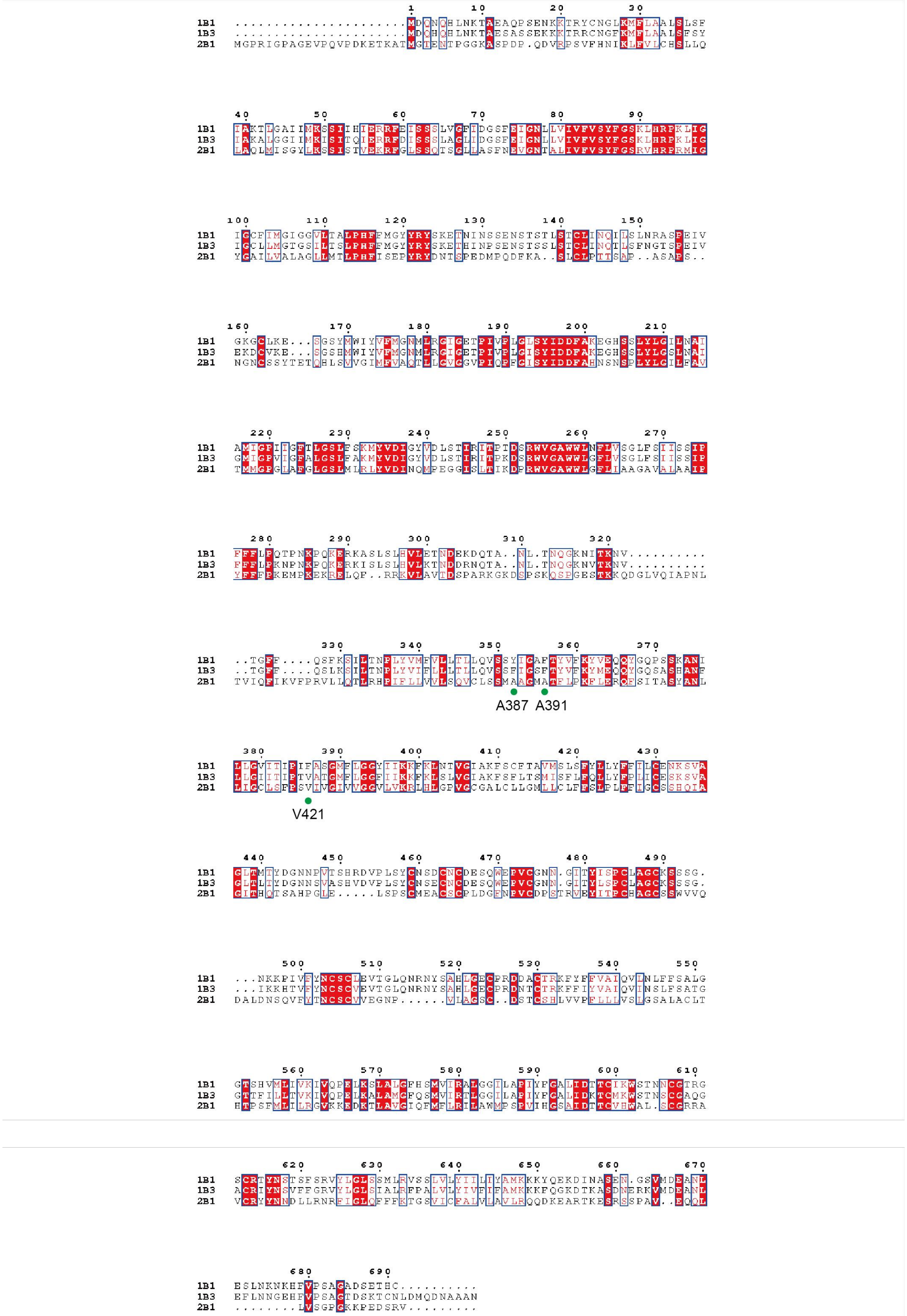

Supplementary Fig. S9 Sequence alignment of OATP1B1、OATP1B3 and OATP2B1. UniProt IDs for the aligned sequences are: Q9Y6L6, Q9NPD5 and O94956.

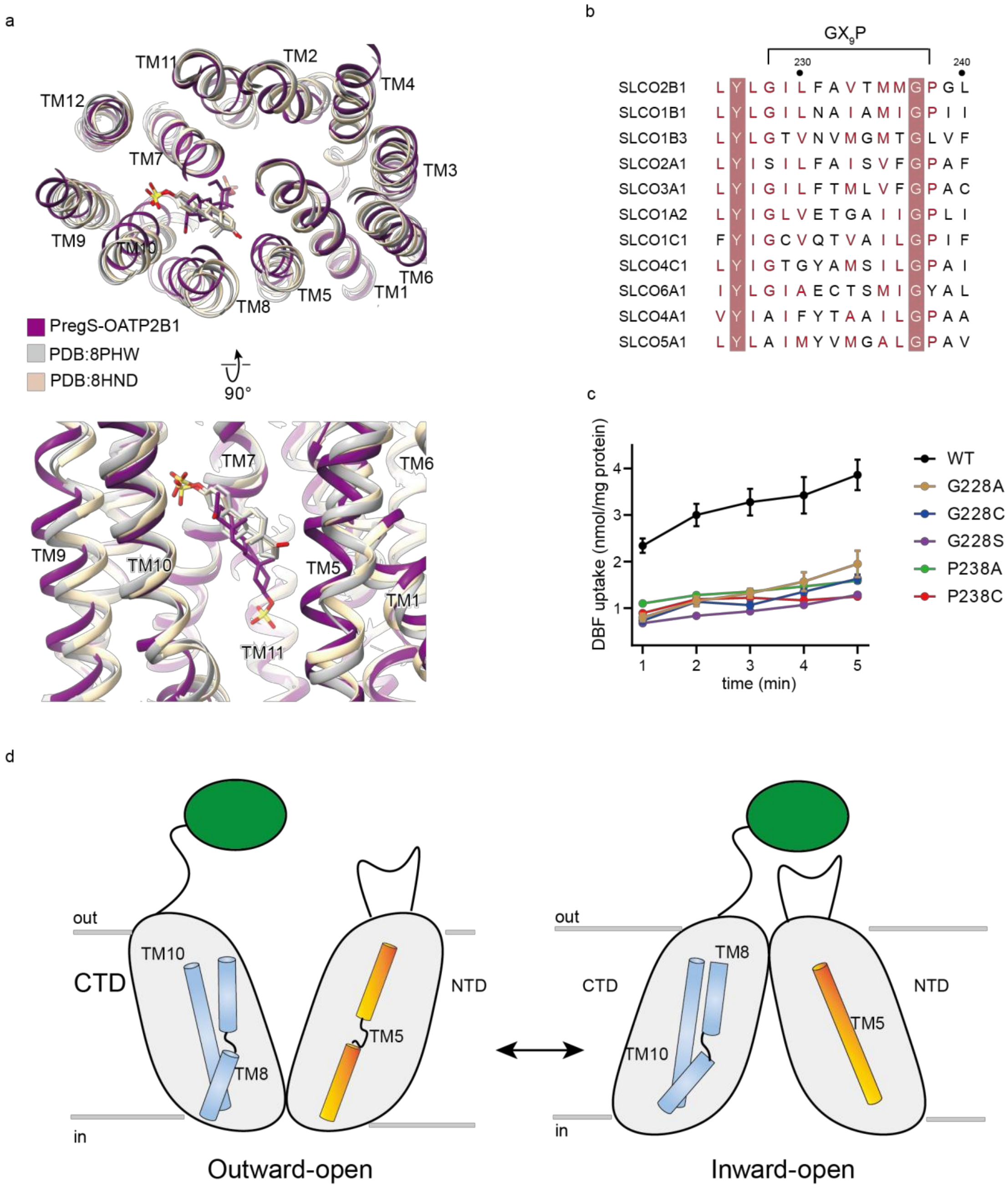

Supplementary Fig. S10 Comparison of substrate recognitions between OATP2B1 and OATP1B1 and the canonical motif. a. Close-up view of the superimposed substrate-binding sites of OATP2B1 and OATP1B1. **b.** A sequence alignment of TM5 from OATP family members. The position of the GX_9P motif is indicated. c**.** DBF uptake activity assay showing reduced activity of OATP2B1 mutants regarding residues within the conserved antiporter motif (mean ± SEM, n=3). **d.** The proposed rocker-switch alternating access model of OATP2B1 during transport.

Table S1 | Cryo-EM data collection, refinement and validation statistics

Protein	OATP2B1-apo (PDB-9VER, EMD-65015)	OATP2B1-PregS (PDB-9VET, EMD-65017)	OATP2B1-DBF (PDB-9VES, EMD-65016)	OATP2B1-Erlotinib (PDB-9VEV, EMD-65019)
**Data collection and processing**				
**Magnification**	81K	81K	81K	81K
**Voltage （kV）**	300	300	300	300
**Electron exposure (e^{-}/Å^2)**	50	50	50	50
**Defocus range (μm)**	-1.0 ~ -2.0	-1.0 ~ -2.0	-1.0 ~ -2.0	-1.0 ~ -2.0
**Magnified pixel size (Å)**	1.055	1.055	1.055	1.055
**Micrographs**	10,163	9,303	12,275	4,888
**Initial particle images (no.)**	6,159,523	5,253,879	4,578,925	3,295,595
**Final particle images (no.)**	71,043	102,273	222,990	71,062
**Symmetry**	C1	C1	C1	C1
**Map Resolution (Å)**	3.63	3.58	3.85	3.62
**FSC threshold**	0.143	0.143	0.143	0.143
**Map resolution range (Å)**	2.8-3.6	2.8-4.0	2.8-3.8	2.8-3.6
**Sharpening B-factor (Å^2)**	140.7	136.3	204.5	150.8
**Model statistics**				
**Model resolution (Å)**	4.0	3.9	4.3	3.9
**Map CC**	0.61	0.59	0.61	0.62
**Number of atoms**	4,164	4,191	4,191	4,193
**B-factor**				
**Protein**	108.25	175.82	210.38	206.07
**Ligand**	---	154.04	280.52	107.53
**R.m.s. deviations**				
**Bond length (Å)**	0.002	0.003	0.003	0.003
**Bond angle (°)**	0.561	0.662	0.661	0.658
**Ramachandran plot**				
**Favored (%)**	96.82	96.63	97.38	96.82
**Allowed (%)**	3.18	3.37	2.62	3.18
**Disallowed (%)**	0.00	0.00	0.00	0.00
**Rotamers Outlier (%)**	0.00	0.00	0.00	0.00
**MolProbity Score**	1.63	1.72	1.66	1.64
**Clash score**	7.96	9.45	10.42	8.29

Materials and Methods

Protein expression and purification

The DNA sequence of human OATP2B1 (Uniprot ID: O94956) was cloned into a pEG BacMam vector. To facilitate affinity chromatography, a Twin-Strep affinity tag, a CFP tag and a 3C protease cleavage site were attached to the N-terminus of OATP2B1. HEK293F cells were employed for expression, cultured in SMM 293-TII Expression Medium (SinoBiological) supplemented with 1% (v/v) fetal bovine serum (FBS). Transient transfection was initiated when cell density reached ~3.0 × 10^6 cells/ml. Plasmids were pre-mixed with polyethylenimine (PEI, Yeasen Biotechnology) at a 1:4 (w/w) ratio prior to transfection. Cells were then maintained at 37 °C in a 5% CO_2 environment for 10 hours. Subsequently, 10 mM sodium butyrate was added to boost protein expression, and the culture temperature was shifted to 30°C for an additional 48 hours before cell harvest.

All steps were performed at 4 °C or on ice unless otherwise specified. The cell pellets were resuspended with buffer A containing 20 mM Tris pH 8.0, 150 mM NaCl, 0.8 µM aprotinin, 2 µg/ml leupeptin, 2 mM pepstatin A and 1 mM phenylmethylsulfonyl fluoride and disrupted via sonication. The crude lysate was first centrifuged at 1,538 ×g for 7 min to remove cellular debris, followed by ultracentrifugation at 124,948 ×g for 1 h to isolate the membrane fraction. The resulting pellet was resuspended in Buffer A, homogenized, and dialyzed against 20 mM Tris-HCl (pH 8.0), 150 mM NaCl for 72 h. The membrane fraction was solubilized with 1% (w/v) n-dodecyl-β-D-maltopyranoside (DDM, Anatrace) and 0.2% (w/v) cholesteryl hemisuccinate (CHS, Anatrace) for 1.5 h. Insoluble material was removed by ultracentrifugation (124,948 ×g, 1 h), and the supernatant was filtered through a 0.45-µm polystyrene membrane before incubation with Strep-Tactin resin for 1 h. Then the resin was washed with 100 column volumes of buffer B consisting of 20 mM Tris pH 8.0, 150 mM NaCl, 0.01% (w/v) lauryl maltose neopentyl glycol (LMNG, Anatrace) and the target protein was eluted using buffer B supplemented

with 5 mM desthiobiotin. The eluted protein was treated with 3C protease for 1 h and further purified by size-exclusion chromatography (SEC) on a Superose 6 10/300 GL column (GE Healthcare) equilibrated with Buffer B. Peak fractions were analyzed by SDS-PAGE and pooled for cryo-EM analysis. For OATP2B1-Erlotinib complex preparation, dialysis was omitted, and solubilization was performed using 1% (w/v) DDM only.

Cryo-EM sample preparation and data acquisition

Protein samples were concentrated to 5-10 mg/mL for cryo-EM grid preparation. For OATP2B1 complexes with ligands, pregnenolone sulfate (PregS, GLPBIO), 4',5'-dibromofluorescein (DBF, MCE), and erlotinib (Selleck) were supplemented at final concentrations of 1 mM, 2 mM, and 100 μM, respectively. A 4-μL aliquot of the protein-ligand mixture was applied to a glow-discharged Quantifoil R2/1 200-mesh gold holey carbon grid, blotted for 3s under 95% humidity at 8°C, and vitrified in liquid ethane using a Vitrobot Mark IV (Thermo Fisher Scientific). Cryo-EM grids were imaged on a Titan Krios electron microscope (Thermo Fisher Scientific) operated at 300 kV, equipped with a K3 direct electron detector (Gatan) and a GIF Quantum energy filter. Movies were acquired using EPU software at a calibrated magnification of 81,000× in super-resolution mode, yielding a pixel size of 0.5275 Å. Images were recorded with a defocus range of −1.0 to −2.0 μm, a total electron dose of 50 $e^-/Å^2$, and a dose rate of ~25 e^-/pixel/s. To improve the pronounced preferential orientation of the particles, we collected additional data by supplementing the samples with 500 μM FOM (Data collection 2) and combined it with the data collected in the absence of FOM (Data collection 1).

Cryo-EM data processing

The data processing pipelines are shown in supplementary information. All the data processing procedures were conducted in CryoSparc v4.5.3[1]. A total of 10,163, 9,303, 12,275, and 4,888 cryo-EM movies were collected for OATP2B1-apo, OATP2B1-PregS, OATP2B1-DBF, and OATP2B1-Erlotinib datasets, respectively.

Movies from two data collections were respectively subjected to gain normalization and beam-induced motion correction using Patch Motion Correction, followed by contrast transfer function (CTF) estimation via Patch CTF. Micrographs with CTF fitting resolutions worse than 4 Å were excluded. Particles were picked out by blob picking, template picking and topaz picking followed by several rounds of 2D classifications. Particles picked by the blob picker, template picker and topaz picker were combined, and duplicate entries (within ~30 Å) were removed. Classes exhibiting clear secondary structure features were used for heterogeneous refinement with three rounds of multi-reference 3D classification using one good reference and three biased maps to exclude undesirable particles.

For the OATP2B1-apo dataset, 880 micrographs from data collection 1 were used for initial particle picking and generation of good ab-initio reconstruction. For the data collection 1, the particles of the best class were refined with non-uniform refinement and local refinement, yielding a 3.38 Å resolution map according to gold-standard Fourier shell correlation (GSFSC) criterion. These particles were subjected to a rebalancing procedure to correct for biased viewing directions. Then, the particles (187,696) were further refined using non-uniform refinement, followed by the local CTF refinement, another non-uniform refinement and local refinement, yielding a 3.75 Å-resolution map. The refined particles were combined with the best class (213,334 particles) from the data collection 2. Heterogeneous refinement was applied to discard poorly aligned particles. The particles from the good class were refined using non-uniform refinement, local refinement, followed by the local CTF refinement and another local refinement. Then the particles were 3D classified without alignment and with masking out the TMD region. The class (71,043 particles) that showed the best resolved TMD region was selected for further local refinement yielding the final EM density map at 3.63 Å resolution.

For the OATP2B1-PregS dataset of the data collection 1, iterative rounds of ab-initio reconstruction were performed to produce a good reconstruction displaying clear

secondary structure features for the subsequent multi-reference heterogeneous refinement. Selected particles (288,119) were used as seeds for the subsequent seed-facilitated guided 3D classifications to enrich good particles. The enriched particles (342,945) were subjected to a rebalancing procedure to correct for biased viewing directions. Then, the particles (148,584) were further refined using non-uniform refinement, followed by the local CTF refinement, another non-uniform refinement and local refinement, yielding a 3.64 Å-resolution map. The combined particles from the two data collections were subjected to heterogeneous refinement to remove the bad particles. And the well-selected particles were further refined using non-uniform refinement and local refinement. Then the particles were 3D classified without alignment and with masking out the TMD region. The class (102,273 particles) that showed the best resolved TMD region was selected for further local refinement, local CTF refinement and another local refinement yielding the final EM density map at 3.58 Å resolution.

For the OATP2B1-DBF dataset of the data collection 1, the OATP2B1-apo map was used for the good reference of heterogeneous refinement. Selected particles (408,476) were further refined using non-uniform refinement, yielding a 3.62 Å-resolution map. The particles were subjected to a rebalancing procedure to correct for biased viewing directions and were further refined using non-uniform refinement. Subsequently, heterogeneous refinement was performed and the particles (232,853) from the good class were refined using non-uniform refinement, producing a 3.77 Å-resolution map. The combined particles from the two data collections were subjected to heterogeneous refinement to remove the bad particles. And the well-selected particles (333,464) were further refined using non-uniform refinement. These particles were subjected to a rebalancing procedure to correct for biased viewing directions. Then, the particles (222,990) were further refined using non-uniform refinement, followed by the local CTF refinement, another non-uniform refinement and local refinement, yielding a 3.85 Å-resolution map.

For the OATP2B1-Erlotinib dataset, iterative rounds of ab-initio reconstruction were performed to produce a good reconstruction for the multi-reference heterogeneous refinement. Successive rounds of 2D classification and heterogeneous refinement were applied to discard poorly aligned particles. Selected particles from the two data collections were combined. Subsequently, these particles (1,011,077) were subjected to a rebalancing procedure to correct for biased viewing directions. Three rounds of heterogeneous refinements were performed and the particles from the good class were refined using non-uniform refinement and local refinement, producing a 3.66 Å-resolution map. Then the particles were 3D classified without alignment and with masking out the TMD region. The class (71,062 particles) that showed the best resolved TMD region was selected for further local refinement yielding the final EM density map at 3.62 Å resolution.

Model building

The AlphaFold-predicted model of OATP2B1 was roughly fitted into the 3D reconstruction map of OATP2B1-apo by UCSF ChimeraX[2]. The Cα backbone and side chains of residues were manually adjusted and rebuilt in Coot[3], followed by iterative refinement in Phenix[4]. The resulting OATP2B1-apo model served as the initial template for the structures of OATP2B1-PregS/DBF/Erlotinib. Geometry constraints for PregS/DBF/Erlotinib were produced by elBow tool in Phenix[4]. The final models for all the datasets were refined against the corresponding maps using phenix.real_space_refine module in Phenix with secondary structure and geometry restraints applied. The model validation was performed using MolProbity[5] and Phenix, with statistics detailed in Supplementary Table 1. All structural figures were prepared using UCSF ChimeraX[2].

Molecular dynamics simulation

Molecular dynamics simulations were performed to elucidate the binding interactions of OATP2B1 with DBF, Erlotinib, and PregS, alongside the evaluation of complex stability. Initial structural models were obtained from cryo-electron microscopy

(cryo-EM) data, followed by iterative conformational optimization. The intact OATP2B1 construct was prepared for simulation by reconstructing unresolved loop segments via homology modeling using SWISS-MODEL[6].

The resulting complexes were embedded in a palmitoyl-oleoyl-phosphatidylcholine (POPC) membrane environment, solvated with TIP3P water, and ionized with K^+ and Cl^- to reach 0.15M ionic strength. Topology construction and system assembly were executed through the CHARMM-GUI[7] platform, employing CHARMM36m[8] and CGenFF[9] parameters. Steepest descent energy minimization was applied to resolve steric clashes. Equilibration proceeded sequentially through six stages with progressively reduced position restraints: initial NVT equilibration for 125 ps (1 fs timestep), followed by NPT equilibration for approximately 1 ns with gradual relaxation of restraints on protein backbone, side chains, and lipids. Production runs of 200 ns were executed in using GROMACS 2023 under periodic boundary conditions with a 2 fs timestep. Temperature was maintained at 303.15 K using the velocity-rescaling thermostat, while pressure was regulated at 1 bar using the C-rescale barostat with semi-isotropic coupling. Long-range electrostatic interactions were calculated using the Particle Mesh Ewald (PME) method, and hydrogen-involved bonds were constrained via the LINCS algorithm. Protein backbone RMSD was calculated using gmx rms after fitting to transmembrane Cα atoms (loops excluded). Ligand RMSD was calculated using gmx rmsdist. For each system, mean ± SD across three independent replicates is reported. All computational procedures were conducted using GROMACS 2023.1[10].

Cell-based transport assay using DBF

For the transport assay, constructs of wild-type and mutant OATP2B1 with N-terminal tag were used. HEK293-T cells cultured in a 24-well plate coated with Poly-L-Lysine (Sigma-Aldrich) were used for transport measurements. Cells were transfected with the plasmid and polyJeT (SignaGen) when density reached ~70-80%. The untransfected cells were used as blank control. 36 h post-transfection, the growth

medium in the plate was removed and cells were washed twice with 500 μL pre-warmed PBS for each well. The cells were then incubated with 200 μL of uptake buffer (142 mM NaCl, 5 mM KCl, 1.2 mM MgSO4, 1.5 mM CaCl2, 1 mM KH2PO4, 5 mM glucose, 12.5 mM HEPES pH 7.4) containing 2.5 μM DBF for 3 min at 37 °C. For the time-course experiment, the uptake was terminated at different time point (1 min, 2 min, 3 min, 4 min, 5 min, 10 min, 15 min and 20 min). For the saturation uptake kinetic assay, uptake was initiated by adding a series of concentrations of DBF (0.03 μM, 0.1 μM, 0.3 μM, 1 μM, 3 μM, 10 μM, 30 μM, 100 μM and 200 μM). After incubation, the uptake buffer was aspirated, and cells were washed three times with 500 μL ice-cold uptake buffer. Subsequently, 250 μL of uptake buffer containing 2% Triton X-100 was added to each well, and the plate was incubated at room temperature for 30 min to lyse cells. Finally, 100 μL of cell lysate was transferred to a 96-well plate, and DBF fluorescence was measured at an excitation/emission wavelength of 532 nm/551 nm.

For the uptake inhibition assay, cells were preincubated with 100 μL of uptake buffer (2×) containing varying concentrations of pregnenolone sulfate (0.06–1200 μM) or erlotinib (0.002–60 μM) at 37°C for 1 min. Uptake was initiated by adding an equal volume of uptake buffer (2×) containing 5 μM DBF and terminated by the addition of ice-cold uptake buffer. Cells with addition of DBF in uptake buffer without compounds were used as a positive control, assumed to possess 100% activity. The cells were then washed and lysed as described above.

For the reverse transport experiments, 2.5 μM DBF was supplied in the uptake buffer to initiate the uptake reaction. After incubation for 3 min, the cells were immediately washed twice with 500 μL of ice-cold assay buffer. Take this moment as zero point of reverse transport and the cells directly lysed to measure fluorescence intensity were regarded as negative controls, assumed the reverse transport to be 0. Cells were then incubated with 200 μL prewarmed assay buffer for different time (15 min, 20 min, 30 min and 60 min) at 37 ℃. The cells were washed twice and lysed as described above.

To examine the effect of extracellular pH, the uptake of DBF at pH 7.4 was compared with the uptake measured in transport buffer of pH 5.5 or pH 6.3, prepared with 12.5 mM 2-(N-morpholino) ethanesulfonic acid (MES). The cells were preincubated at pH 7.4, followed by measurement of the uptake of DBF in buffer of pH 5.5, 6.3, or 7.4 to generate a proton gradient. After the incubation of 3 min, the cells were washed and lysed as described above.

For calibrating the expression of OATP2B1 variants, cell surface biotinylation was performed to normalize OATP2B1 variant expression levels. Wild-type and mutant OATP2B1 constructs with a N-terminal Flag and CFP tag were transiently transfected into HEK293F cells. After 60 h, cells were harvested, washed with cold PBS, and subjected to cell-surface biotinylation using 0.8 mg·mL^{-1} sulfo-NHS-SS-biotin (Glpbio) at 4 °C for 30 min. Cells were then quenched with 100 mM glycine in cold PBS and lysed in lysis buffer (20 mM Tris pH 8.0, 150 mM NaCl, 1% DDM, 0.2% CHS, and protease inhibitors) at 4 °C for 1 h. After centrifugation at 40,000×g for 30 min, supernatants were incubated with Strep-Tactin resin. Beads were washed with ice-cold wash buffer and incubated with 5× loading buffer containing 5% β-mercaptoethanol. Samples were separated by SDS-PAGE and analyzed by western blotting. PVDF membranes were blocked in 5% milk, then probed with anti-CFP (Proteintech, 1:5000) and anti-Na^+/K^+-ATPase (Abclonal, 1:10,000) primary antibodies overnight at 4 °C. After incubation with HRP-conjugated secondary antibody (Proteintech, 1:10,000), signals were detected using a ChemiDoc MP Imager (Bio-Rad).

References

1 Punjani, A., Rubinstein, J. L., Fleet, D. J. & Brubaker, M. A. cryoSPARC: algorithms for rapid unsupervised cryo-EM structure determination. *Nat Methods* **14**, 290-296 (2017). https://doi.org:10.1038/nmeth.4169

2 Goddard, T. D. *et al.* UCSF ChimeraX: Meeting modern challenges in visualization and analysis. *Protein Sci* **27**, 14-25 (2018). https://doi.org:10.1002/pro.3235

3 Emsley, P. & Cowtan, K. Coot: model-building tools for molecular graphics.

Acta Crystallogr D Biol Crystallogr **60**, 2126-2132 (2004). https://doi.org:10.1107/S0907444904019158

4 Afonine, P. V. *et al.* Real-space refinement in PHENIX for cryo-EM and crystallography. *Acta Crystallogr D Struct Biol* **74**, 531-544 (2018). https://doi.org:10.1107/S2059798318006551

5 Chen, V. B. *et al.* MolProbity: all-atom structure validation for macromolecular crystallography. *Acta Crystallogr D Biol Crystallogr* **66**, 12-21 (2010). https://doi.org:10.1107/S0907444909042073

6 Waterhouse, A. *et al.* SWISS-MODEL: homology modelling of protein structures and complexes. *Nucleic Acids Res* **46**, W296-W303 (2018). https://doi.org:10.1093/nar/gky427

7 Jo, S., Kim, T., Iyer, V. G. & Im, W. CHARMM-GUI: a web-based graphical user interface for CHARMM. *J Comput Chem* **29**, 1859-1865 (2008). https://doi.org:10.1002/jcc.20945

8 Klauda, J. B. *et al.* Update of the CHARMM all-atom additive force field for lipids: validation on six lipid types. *J Phys Chem B* **114**, 7830-7843 (2010). https://doi.org:10.1021/jp101759q

9 Best, R. B. *et al.* Optimization of the additive CHARMM all-atom protein force field targeting improved sampling of the backbone phi, psi and side-chain chi(1) and chi(2) dihedral angles. *J Chem Theory Comput* **8**, 3257-3273 (2012). https://doi.org:10.1021/ct300400x

10 van der Spoel, D. *et al.* GROMACS: Fast, flexible, and free. *Journal of Computational Chemistry* **26**, 1701-1718 (2005).
